# Support Vector Machine Identification of Small Molecule Binders to an Understudied Allosteric Site of SARS‐CoV‐2 Mpro for Next‐Generation PROTAC‐Based Therapeutics

**DOI:** 10.1002/ardp.70169

**Published:** 2025-12-13

**Authors:** Enrico Mario Alessandro Fassi, Nedra Mekni, Marco Albani, Sabine Maehrlein, Annabelle Carolin Weldert, Tanja Schirmeister, Thierry Langer, Giovannf razioso

**Affiliations:** ^1^ Department of Pharmaceutical Sciences Università degli Studi di Milano Milano Italy; ^2^ Department of Pharmaceutical Sciences, Division of Pharmaceutical Chemistry University of Vienna Vienna Austria; ^3^ Department of Medicinal Chemistry, Institute of Pharmaceutical and Biomedical Sciences Johannes Gutenberg‐University Mainz Germany

**Keywords:** 3CL protease, covid‐19, drug design, machine learning, Mpro, PROTAC

## Abstract

The emergence of the severe acute respiratory syndrome coronavirus 2 (SARS‐CoV‐2) has underscored the urgent need for novel antiviral strategies. One of the primary targets of interest is the SARS‐CoV‐2 main protease (Mpro), which plays a crucial role in viral replication. Building on our prior work involving machine learning (ML)‐based virtual screening for potential Mpro inhibitors, we sought to experimentally validate top‐ranked candidates. Microscale thermophoresis (MST) was used to assess the binding affinity, leading to the identification of three promising hits from a library of 180 compounds. Notably, one compound demonstrated high‐affinity binding to SARS‐CoV‐2 Mpro (*K*_d_ = 2.8 ± 0.9 µM). However, enzymatic assays revealed that none of the hit compounds inhibited the activity of the protease, suggesting a non‐competitive binding. Docking and molecular dynamics (MD) simulations allowed to identify an accessory site in which the compounds exhibited stable interactions. These findings suggest that the identified compounds may serve as a starting point for the rational design of degradation‐inducing strategies, such as proteolysis‐targeting chimeras (PROTACs), targeting SARS‐CoV‐2 Mpro, and highlight the value of integrating ML‐driven discovery with biophysical and computational validation in antiviral drug development.

## Graphical abstract

An SVM‐based ML model identified 180 potential SARS‐CoV‐2 Mpro inhibitors. MST experiments confirmed three high‐affinity binders, with one showing *K*_d_ = 2.8 ± 0.9 μM. These compounds are non‐competitive, suggesting binding to a novel allosteric site. This discovery enables rational PROTAC design for Mpro degradation, demonstrating the power of integrating ML, biophysics, and computation in antiviral drug discovery.

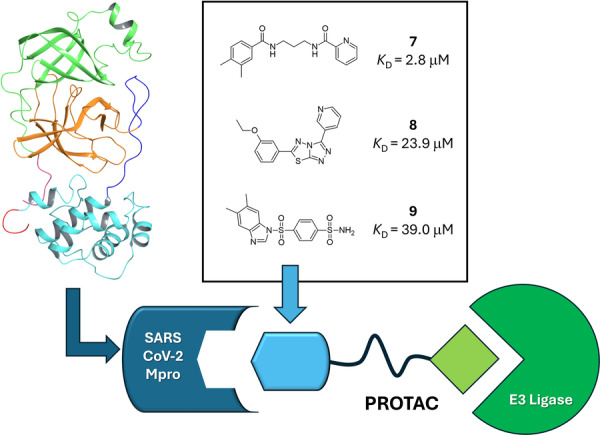

## 1 Introduction

The global coronavirus disease 2019 (COVID‐19) outbreak has intensified the demand for potent antiviral agents [1]. A key enzyme in the severe acute respiratory syndrome coronavirus 2 (SARS‐CoV‐2) replication process is represented by main protease (Mpro), also known as 3CL protease, which gained considerable attention as a drug target [2]. Its therapeutic relevance is reinforced by its high sequence conservation across β‐coronaviruses and its lack of close homologs in the human proteome, minimizing the potential off‐target side effects [3]. Additionally, given its essential role in the viral life cycle, Mpro has become a central focus for the development of selective inhibitors with clinical potential [4]. In 2022, the FDA approved the combination of ritonavir (an anti‐HIV drug) and nirmatrelvir (also known as PF‐07321332) with the commercial name Paxlovid (Pfizer Inc., New York, NY, USA), for treating severe COVID‐19, underscoring the importance of incorporating Mpro inhibitors into the therapeutic arsenal against the virus [5, 6].

**Figure 1:**
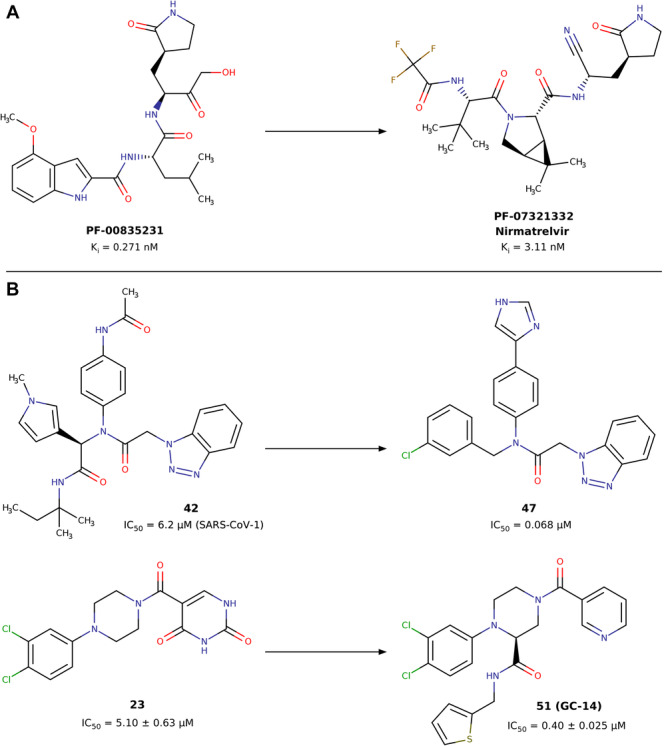
Covalent and non‐covalent inhibitors of the SARS‐CoV‐2 Mpro. (A) The covalent inhibitor nirmatrelvir (also known as PF‐07321332). (B) Non‐covalent inhibitors: compound **47** (CCF981) derived from optimization of lead **42** (ML300), and hit compound **23** (MCULE‐5948770040), selected as lead compounds in the rational design of highly potent and selective SARS‐CoV‐2 Mpro inhibitors **51** (**GC‐14**) [9, 10, 11].

SARS‐CoV‐2 Mpro is a homodimeric cysteine protease resembling chymotrypsin, featuring a non‐standard catalytic dyad comprising Cys145 and His41 [2]. This dyad forms crucial hydrogen bonds with a water molecule necessary for hydrolyzing the amide bond of substrates [7]. SARS‐CoV‐2 Mpro, along with the SARS‐CoV‐2 papain‐like protease (PLpro), plays a pivotal role in processing viral polyproteins, acting during the initial phases of viral replication within host cells [8]. Inhibiting one or both enzymes represents a promising therapeutic approach to impede the proliferation of SARS‐CoV‐2 within infected cells. The literature documents a variety of compounds containing diverse warheads, demonstrating either non‐covalent or covalent activity against SARS‐CoV‐2 Mpro (Figure 1) [12].

Covalent inhibitors exert their inhibitory effects on SARS‐CoV‐2 Mpro by establishing covalent bonds with specific nucleophilic residues in the catalytic site, notably the cysteine residue at position 145 (Cys145) [13]. On the other hand, non‐covalent inhibitors competitively bind to the catalytic site through classical hydrogen bonds, electrostatic, and non‐polar interactions. To date, nirmatrelvir remains the only approved peptide‐based therapeutic for COVID‐19 that exerts its antiviral effect through covalent inhibition of the SARS‐CoV‐2 Mpro [14]. Conversely, most non‐covalent Mpro inhibitors are still in the early stages of development [15, 16, 17]. For example, compound **47** (CCF981, Figure 1B) exhibited an IC_50_ of 68 nM against SARS‐CoV‐2 Mpro and demonstrated EC_50_ values of 0.50 and 0.56 µM in virus‐infected VeroE6 cells, measured by cytopathic effect (CPE) inhibition and plaque reduction assays, respectively. Similarly, compound **51** (GC‐14, Figure 1B), a 1,2,4‐trisubstituted piperazine optimized through structure‐based design, showed a significant antiviral activity in vitro, with an EC_50_ of about 1.1 µM in assays using infected cells and an IC_50_ of about 0.40 µM against Mpro [9, 10, 11]. Therefore, there is a pressing need to intensify research efforts toward the development of non‐covalent inhibitors. These compounds show potential for addressing the challenges linked to peptide drugs, providing better pharmacological features like lower immunogenicity and increased stability [18].

Considering a previous study in which a support vector machine (SVM)‐based machine learning (ML) model was developed to identify potential SARS‐CoV‐2 Mpro inhibitors [19], this work presents the next phase of our drug discovery pipeline, focusing on biophysical validation of the computational predictions. The SVM classifier was trained to distinguish active from inactive compounds based on physicochemical properties rather than structural similarity, offering an advantage in scenarios where mechanistic understanding is limited [19]. In the field of drug design, SVM has proven effective in various drug design applications, including predicting drug metabolism, identifying P‐glycoprotein substrates, and evaluating blood–brain barrier permeability [20]. After extensive model optimization and cross‐validation, 180 top‐ranked commercially available compounds were selected and experimentally tested through microscale thermophoresis (MST). Despite exhibiting promising binding profiles in those biophysical assays, the most promising compounds failed to inhibit enzymatic activity, leading to the hypothesis that their binding does not occur in the active site of SARS‐CoV‐2 Mpro.

Consequently, in this study, we report on the computational studies aimed at the identification of the accessory site of SARS‐CoV‐2 Mpro in which the selected compounds exhibit stable interactions. Specifically, using a combination of pocket detection algorithms, molecular docking studies, and molecular dynamics (MD) simulations, we identified a pocket that has received limited attention in the literature, further suggesting its potential as a target for antiviral drug development. In fact, while most therapeutic efforts have focused on targeting the catalytic site of SARS‐CoV‐2 Mpro, accessory sites represent promising targets for designing alternative antiviral drugs with potentially lower resistance risk [21]. Depending on their position, the external sites may influence the catalytic activity of the enzyme, in which case they are referred to as “allosteric sites.” In contrast, when the accessory sites do not alter enzymatic activity, they can serve as strategic anchoring points for designing molecular glues such as PROteolysis‐TArgeting Chimeras (PROTACs). PROTACs represent a novel class of promising drugs inducing the selective degradation of disease‐associated proteins, with significant applications in different fields. PROTACs are heterobifunctional molecules that simultaneously bind a target protein and an E3 ubiquitin ligase, facilitating the formation of a ternary complex [22, 23]. The E3 ligase itself is in complex with an activated ubiquitin‐loaded E2 enzyme, and this close association facilitates the (poly)‐ubiquitination of the target protein, marking it for degradation by the 26S proteasome [24]. In particular, they consist of a “warhead” group that binds to a target protein and an ”anchor” group that binds to the substrate‐binding domain of an E3 ubiquitin ligase, connected by a more or less flexible linker [23, 25].

Several studies have reported the development of PROTACs targeting the SARS‐CoV‐2 Mpro. Notably, Alugubelli and co‐workers designed PROTAC degraders derived from previously characterized reversible covalent SARS‐CoV‐2 Mpro inhibitors (MPI8 and MPI29), conjugated to a CRBN E3 ligase anchor, which reported to reduce Mpro levels in human cells through a CRBN‐dependent, proteasome‐mediated mechanism [26, 27, 28]. Among these, MPD2 demonstrated potent degradation of Mpro in both 293T cells and SARS‐CoV‐2‐infected A549‐ACE2 cells, exhibiting antiviral activity across multiple viral strains, including nirmatrelvir‐resistant variants, with an EC_50_ of approximately 492 nM [27]. In addition, a series of indomethacin‐based PROTACs recruiting either VHL or CRBN E3 ligases were developed, showing broad‐spectrum antiviral effects, with enhanced antiviral activity compared with indomethacin alone, achieving low micromolar to nanomolar EC_50_ values [29].

Collectively, these findings establish that PROTACs can be rationally designed to selectively degrade SARS‐CoV‐2 Mpro, offering a promising therapeutic strategy that may overcome challenges such as drug resistance and toxicity associated with conventional inhibitors. In fact, unlike conventional orthosteric ligands that function by occupying the active site to block activity, PROTACs act through a catalytic mechanism, eliminating the protein entirely. This catalytic mechanism allows PROTACs to exert their effects at lower concentrations compared with traditional inhibitors, which often require higher systemic exposure for therapeutic efficacy [30, 31].

## 2 Results and Discussion

### 2.1 Selection of the SARS‐CoV‐2 Mpro Inhibitors

In a previous study, published by some of us, an ML‐based virtual screening model was developed to identify novel SARS‐CoV‐2 Mpro inhibitors [19]. The model architecture employed a supervised classification framework using an SVM with a radial basis function kernel [32] and was trained on the PostEra COVID‐19 Moonshot dataset, an open‐access collection of small molecules annotated with experimentally measured IC_50_ values [33]. In this work, the trained model (see Section 4 for details) was employed to perform a virtual screening of a commercial compound library comprising approximately two million small molecules obtained from different vendors such as MolPort, Asinex, and ChEMBL. Out of 200 compounds predicted as potential Mpro inhibitors, 180 were selected for experimental validation using MST due to their commercial availability.

### 2.2 Biophysical Experiments

**Figure 2:**
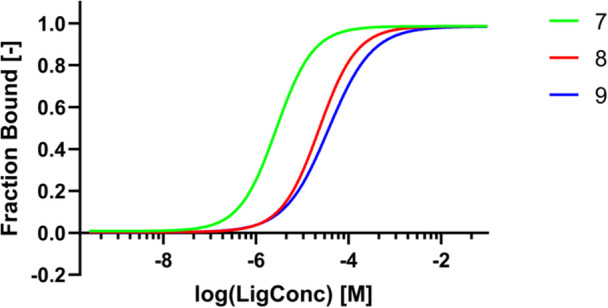
MST curves acquired by SARS‐CoV‐2 Mpro incubated with scaling concentrations of compounds **7** (green), **8** (red), and **9** (blue) in comparison with each other, using the Monolith NT.115 instrument. The curves were normalized by fraction bound. Two independent experiments were performed to compute the *K*_d_ curve.

Following an experimental protocol previously reported by us [34, 35], MST experiments were conducted to estimate the *K*_d_ values of the 180 compounds selected from the SVM‐based virtual screening model against the SARS‐CoV‐2 Mpro, quantifying interactions between the designed ligands and the protein binding site [19]. This biophysical technique allows for the analysis and measurement of molecular interactions between two entities under solution equilibrium conditions, avoiding the need for the sample immobilization which could potentially interfere with binding, as in the case of surface plasmon resonance method [36]. Initially, binding check experiments were accomplished using a ligand fixed concentration of 50 µM (see Section 4 for details). This initial step was designed to determine which small molecules are capable of binding to the SARS‐CoV‐2 Mpro protein at the tested concentration. Out of the 180 compounds screened, 17 showed positive results in the binding assay and were chosen for further analysis of their binding affinities. The chemical structures of the 17 compounds are provided in the Supporting Information S1: Table S1. To obtain a complete *K*_d_ curve, a fixed concentration of labeled SARS‐CoV‐2 Mpro enzyme was mixed with 16 1:1 serial dilutions of the compounds, ranging in concentration from 250 µM to 7.6 nM (see Section 4 for details). Three out of the 17 tested compounds (**7**, **8**, and **9**) displayed a clear *K*_d_ curve in two independent replicas. In the remaining cases, a significant number of outlier points prevented the computation of the complete affinity curve making impossible to estimate the *K*_d_ value for those compounds. Among the compounds showing clear *K*_d_ curves, the compound **7** showed the highest affinity for SARS‐CoV‐2 Mpro enzyme displaying a *K*_d_ of 2.8 ± 0.9 µM, followed by **8** (*K*_d_ = 23.9 ± 7.4 µM), and **9** (*K*_d_ = 39.0 ± 23.4 µM) as detailed in Table 1 and shown in Figure 2. The summary table of the MST assays experimental conditions performed, and the detailed MST *K*_d_ curve of each compound are available in the Supporting Information S1: Table S2 and Figure S1, respectively. In the case of compound **7**, the first three concentration points (corresponding to 250, 125, and 62.5 µM, respectively) were excluded by fitting, since they seem to be part of another binding curve observed in the high micromolar range, probably due to its nonspecific binding on the SARS‐CoV‐2 Mpro protein (Supporting Information S1: Figure S2).

**Table 1:** ID number, *K*_d_ values, and chemical structure of compounds that displayed a clear *K*_d_ curve in MST.

Compound ID	*K*_d_ ± *K*_d_ confidence (µM)	Structure
**7**	2.8 ± 0.9	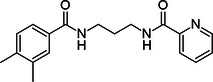
**8**	23.9 ± 7.4	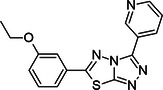
**9**	39.0 ± 23.4	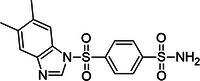

### 2.3 Enzymatic Assays

The three compounds identified as binders in the MST assay were tested for their inhibitory properties against SARS‐CoV‐2 Mpro at a 20 µM concentration (see Section 4 for details). However, none of the compounds caused a significant reduction in the enzyme's activity (Table 2 and Supporting Information S1: Figure S3).

**Table 2:** Residual activity of compounds screened against SARS‐CoV‐2 Mpro at 20 µM in enzymatic assay.

Compound ID	Residual activity (%)	Error
**7**	104.66	3.03
**8**	100.80	2.94
**9**	106.45	4.02

These results suggest that, at the tested concentration, the compounds likely do not bind within the active site as orthosteric or allosteric ligands. Consequently, to investigate alternative binding sites, we conducted further computational studies, including the identification of protein accessory sites, molecular docking calculations, and MD simulations.

### 2.4 Accessory Sites Identification

**Figure 3:**
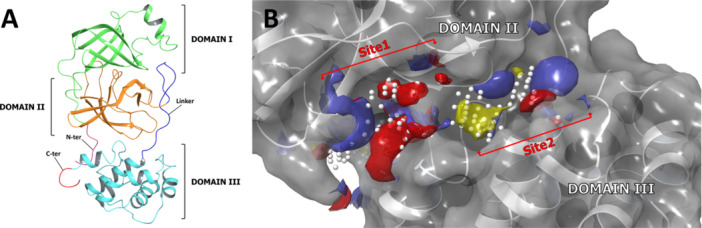
General overview of Mpro structure. Representation (A) of the three structural domains of SARS‐CoV‐2 Mpro (Domain I: green, Domain II: orange, Domain III: cyan) and of (B) the two allosteric sites (Site1 and Site2), located at the interface between Domain II and Domain III, identified with SiteMap.

The SiteMap tool, available in Maestro software (Schrödinger LLC, New York, NY, USA), was used to identify potential binding sites on the crystal structure of the target enzyme (PDB accession code: 6W63), which were ranked based on their “SiteScore” values (Table 3). Notably, the SARS‐CoV‐2 Mpro structure comprises three domains: Domains I (residues 8‐101) and II (residues 102–184) consist of antiparallel β‐barrel structures and serve as catalytic domains, while Domain III (residues 201–303) comprises five α‐helices and is responsible for enzyme dimerization (Figure 3A) [3].

**Table 3:** Potential binding sites identified by SiteMap, along with their SiteScore, size, and volume values.

Binding site	SiteScore	Size (Å^2^)	Volume (Å^3^)
Site1	0.83	74	183
Active Site	0.81	46	129
Site2	0.80	51	159
Site3	0.56	32	61
Site4	0.51	16	66

SiteMap identified a total of five potential binding sites and correctly recognized the active site as a promising binding region. However, this was not ranked as the most favorable site (Table 3). On the other hand, two sites (Site1 and Site2) were found to be adjacent to each other and located at the interface between Domain II and Domain III, on the opposite side of the active site (Figure 3B). These two sites showed SiteScore values slightly higher (Site1) or comparable (Site2) to that of the active site (Table 3). Site3 and Site4 (Table 3), located within Domain II and Domain III, respectively, showed considerably low SiteScore values and were found to be very small and shallow, lacking well‐defined pockets (Supporting Information S1: Figure S4). Thus, they were excluded from the following docking phase. Consequently, Site1 and Site2 were selected for molecular docking calculations.

It was interesting to note that these sites were also investigated by Günther and co‐workers, whose employed X‐ray crystallography to screen over 5000 compounds, either approved drugs or those in clinical trials, identifying 37 compounds targeting SARS‐CoV‐2 Mpro, primarily at the active site, but also revealing two additional binding sites [37]. Specifically, Site1 in our study was targeted by only a single compound, AT7519 (a CDK inhibitor) [38], for which the crystal structure has also been published (PDB accession code 7AGA) [37]. However, AT7519 displayed weak antiviral activity (EC₅₀ = 25.2 μM), and its inhibitory effect on SARS‐CoV‐2 Mpro enzymatic activity was not experimentally evaluated [39]. The absence of these data prevents determining whether this site can be classified as an allosteric pocket. Indeed, Samrat and colleagues reported on the identification of three niclosamide derivatives as potent non‐competitive inhibitors of SARS‐CoV‐2 Mpro, with IC₅₀ values comparable to that of the known covalent inhibitor boceprevir, thereby suggesting a potential allosteric mechanism of inhibition [40, 41]. Through computational studies, including molecular docking and MD simulations, the authors provided an evidence that the binding site of these compounds could be the same site targeted by the compound AT7519 [37, 41].

### 2.5 Docking and MD Simulations

The residues defining the Site1 and Site2 pockets (see Section 4 for details) were selected to construct the receptor grid, ensuring that the bounding box fully encompassed the spatial volume of both binding sites, thereby capturing all relevant interactions for subsequent docking simulations. Subsequently, molecular docking calculations were conducted for the three ligands targeting the SARS‐CoV‐2 Mpro using the Extra Precision (XP) mode of the Glide module implemented in the Maestro software suite (Release 2025‐1, Schrödinger LLC, New York, NY, USA) [42]. Interestingly, the obtained docking scores are in agreement with the experimental binding affinities determined by MST, providing a supporting evidence that the identified allosteric pocket may represent the actual binding site of these compounds. Specifically, compound **7** exhibited the most favorable docking score of –3.440 kcal/mol, consistent with its highest binding affinity, while compounds **8** and **9** showed lower scores of –3.279 and –2.026 kcal/mol, respectively. For each compound, the top‐ranked binding pose was subjected to three independent 500 ns MD simulations using the Desmond module integrated within the Maestro software suite (Release 2025‐1, Schrödinger LLC, New York, NY, USA), with the goal of evaluating the stability and binding mode of the ligands over the simulation time within the identified allosteric site (see Section 4 for details).

The MD simulations revealed that all three ligands remained stably bound within the identified allosteric site of SARS‐CoV‐2 Mpro throughout the entire simulation period (Supporting Information S1: Figure S5). Notably, compound **7** exhibited the most significant rearrangement from its initial docking pose, as indicated by the highest RMSD peaks before stabilizing into a conformation that maintained persistent interactions with key residues of the allosteric site (Supporting Information S1: Figure S5).

**Figure 4:**
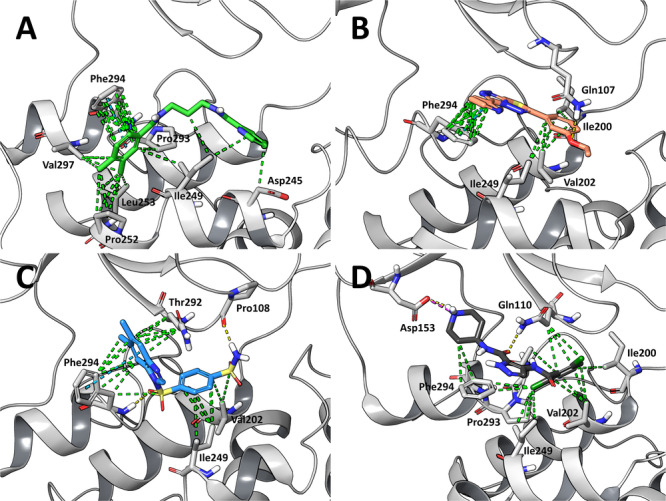
Binding modes of selected compounds. Representative structure from the most populated cluster of (A) compound **7** (green sticks), (B) compound **8** (light red sticks), and (C) compound **9** (light blue sticks) in complex with SARS‐CoV‐2 Mpro (white cartoon) after 500 ns of MD simulation. Hydrophobic, H‐bond, and π–π interactions are represented as green, yellow, and cyan dashed lines, respectively. (D) Crystal structure of AT7519 (dark gray sticks) in complex with SARS‐CoV‐2 Mpro (white cartoon), with PDB accession code 7AGA.

To derive a reliable computational model of the ligand/Mpro complexes, we conducted a cluster analysis of the 1.5 µs MD simulations. The representative structures from the most populated clusters for compounds **7**, **8**, and **9** are shown in Figure 4. For compound **7**, which represents about the 49% of the conformational ensemble explored, the interaction network with SARS‐CoV‐2 Mpro is predominantly characterized by a π–π stacking interaction with Phe294, along with several hydrophobic contacts involving surrounding nonpolar residues that form a hydrophobic pocket (Ile249, Pro252, Leu253, Pro293, Phe294, and Val297), which collectively contribute to the stabilization of the entire ligand within the allosteric binding pocket (Figure 4A). The representative structure of compound **8**, which constitutes the 64.8% of the sampled conformational ensemble, predominantly exhibits hydrophobic interactions, although fewer than those observed for compound **7**. Notably, it forms interactions with Phe294 (including a π–π stacking interaction), Ile200, Val202, and Ile249. Additionally, a hydrogen bond with Gln107 is observed (Figure 4B). This limited interaction profile may account for the lower binding affinity experimentally determined by MST (Figure 2). Compound **9**, representing about the 86% of the sampled conformational ensemble, forms two hydrogen bonds with the backbone amine of Phe294 and the carboxyl group of Pro108 and an important hydrophobic interaction network, including a π–π stacking interaction with Phe294 (Figure 4D). Unexpectedly, despite these interactions, it shows the lowest binding affinity among the three compounds based on MST data (Table 1).

The binding modes of the compounds were compared with the crystallographic pose of AT7519 (Figure 4D). Superimposition of the complexes (Supporting Information S1: Figure S6) revealed that none of our compounds induce a significant conformational change in the apo protein via interaction with Asp153, unlike what is observed for AT7519 [37]. This interaction is critical since the conformational change induced by AT7519 facilitates the formation of strong interactions between Asp153 and Arg298, including H‐bonds and salt bridges and it is important to note that Arg298 is essential for dimerization [37, 43]. In particular, its replacement with Ala residue shifts the spatial arrangement between the dimerization and catalytic domains, thereby altering the oxyanion hole and weakening the S1 pocket through perturbations of the N‐terminal structure [43]. This difference may explain why compounds **7**, **8**, and **9** may have no effect on the catalytic activity of SARS‐CoV‐2 Mpro at the tested concentration of 20 µM (Table 2). However, it is also noteworthy that AT7519 exhibits only low antiviral activity against SARS‐CoV‐2 replication in Vero E6 cells, with an EC_50_ of 25.16 µM, suggesting that the binding site for AT7519 (and likely our compounds as well) may have limited influence on the enzyme's physiological function.

The generation of these computational binding models enabled a deeper understanding of the molecular regions of the compounds most involved in the interactions with SARS‐CoV‐2 Mpro, as well as those predominantly solvent‐exposed. This insight is crucial for guiding the strategic addition of functional moieties needed for the design of potential PROTACs.

### 2.6 Rational Design of Protacs

**Figure 5:**
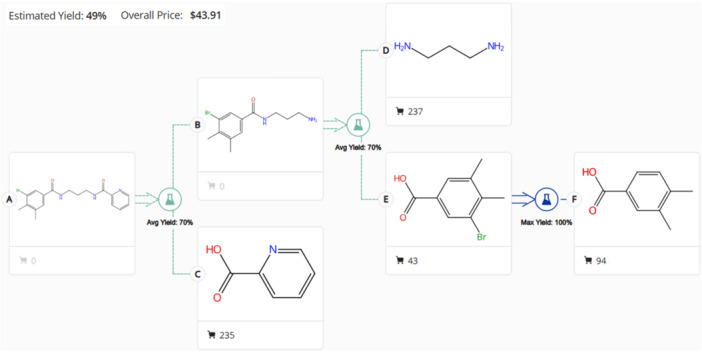
Retrosynthetic analysis of compound **7** intermediate relevant to PROTAC synthesis. The black cart indicates that the product is commercially available and the number of vendors. The blue reaction symbol indicates that the reaction has been already published in literature, while the green one indicates that the reaction is possible, although do not already accomplished using the specific reagents. The scheme was generated through the “retrosynthesis tool” available on CAS‐SciFinder web platform.

Here, we propose the design of potential warheads for PROTAC development targeting SARS‐CoV‐2 Mpro, based on the compounds we have identified that exhibit a binding affinity to the target protein in the micromolar range, as shown by MST experiments (Table 1). The proposed warheads were rationalized based on their favorable binding modes observed after three independent replicas of 500 ns‐long MD simulations (Figure 4), and considering the synthetic feasibility. This latter point was verified through the retrosynthesis tool implemented into CAS SciFinder (Columbus, OH, USA). Based on the chemical structures of the compounds, classical click‐type reactions to generate the corresponding PROTACs can be accomplished, primarily via the Sonogashira cross‐coupling and, in the case of compound **8**, also through the Huisgen cycloaddition. Both the Sonogashira cross‐coupling of terminal alkynes with aryl iodides, bromides, or chlorides (not preferable, as it operates under more forcing conditions), and the Huisgen cycloaddition between an azide and an alkyne are widely used for the introduction of alkynyl or 1,2,3‐triazole linkers, respectively, in the assembly of PROTACs [44, 45]. Given the significantly higher affinity observed for compound **7**, as well as its better synthetic feasibility, this molecule is undoubtedly the most promising among the three to serve as a starting point for PROTAC design (Table 4). Therefore, compound **7** is described in detail here (Figure 5), while the characterization of the other compounds are described in a dedicated section of the Supporting Information, and represented in Supporting Information S1: Figures S7–S10.

**Table 4:** Proposed optimal linker position for rational design of potential PROTACs targeting SARS‐CoV‐2 Mpro based on the compound **7**, indicated by red “R” labels.

Compound 7	
Original structure	Intermediate relevant to PROTAC synthesis
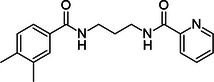	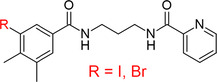

The analysis of the compound **7** binding mode within the accessory pocket reveals that the free meta‐position of the dimethyl‐benzene group is the most favorably oriented for linker attachment (Figure 4A), as it is predominantly solvent‐exposed. Hence, we recommend its substitution with iodides or bromides in the rational design of a PROTAC applying the Sonogashira cross‐coupling reaction (Table 4). The retrosynthetic approach suggested that bromine is the preferred halogen substituent, with an estimated yield of 49% and an estimated cost of $44 per 100 g, versus a 29% estimated yield and a cost of $73 per 100 g for iodine. In particular, the commercially available 3,4‐dimethylbenzoic acid was first brominated to yield 3‐bromo‐4,5‐dimethylbenzoic acid. This intermediate was then amidated with commercially available 1,3‐propanediamine to produce N‐(3‐aminopropyl)‐3‐bromo‐4,5‐dimethylbenzamide. Subsequently, a second amidation with commercially available picolinic acid afforded N‐(3‐(3‐bromo‐4,5‐dimethylbenzamido)propyl)picolinamide (Table 4), which represents the final compound to be used as substrate for the subsequent Sonogashira cross‐coupling reaction to synthesize the PROTAC. The retrosynthesis scheme is reported in Figure 5.

## 3 Conclusions

The integrated computational and biophysical approaches employed in this study demonstrated a promising strategy for the discovery of small‐molecule binders targeting SARS‐CoV‐2 Mpro, with potential application in the rational design of innovative targeted protein degradation therapeutics, such as PROTACs. In particular, using an SVM‐based virtual screening, we prioritized 180 candidate binders covering a huge chemical space, comprising approximately two million compounds retrieved form MolPort, Asinex, and ChEMBL database. The subsequent validation, using MST, identified three promising hits with binding affinities in the low micromolar range, with compound **7** exhibiting a *K*_d_ value of 2.8 ± 0.9 µM. The lack of enzymatic inhibition at the tested concentration of 20 µM strongly suggested a non‐competitive binding mode, prompting us to investigate the possibility of alternative interaction sites on the protein surface. Further computational analyses revealed consistent and energetically favorable interactions within an accessory pocket, spatially distinct from the catalytic site. Finally, we proposed the structure of next‐generation PROTACs directed against SARS‐CoV‐2 Mpro based on the scaffolds of the compounds here disclosed. This strategy not only expands the chemical space for antiviral drug development but also holds potential for improved safety profiles by reducing off‐target effects and associated toxicities typically observed with conventional inhibitors.

## 4 Materials and Methods

### 4.1 SVM Model

The model was trained on the PostEra COVID‐19 Moonshot dataset, an open‐access collection of small molecules first released in March 2020, in which each compound is annotated with an experimentally measured IC_50_ [33]. All compounds were imported as SMILES and converted into 3D molecular structures using RDKit (www.rdkit.org/docs/api-docs.html). This preprocessing included the addition of explicit hydrogens and the generation of low‐energy conformers using the ETKDG algorithm [46]. We then computed 1444 one‐ and two‐dimensional descriptors with PaDEL‐Descriptor, capturing topological indices, electronic features, atomic‐property distributions, and physicochemical parameters [47]. Given the high dimensionality of the descriptor space relative to the number of compounds, and to prevent overfitting during model training, we implemented a feature selection strategy. This approach combined random forest (RF) importance ranking with recursive feature elimination and fivefold cross‐validation (RF‐RFE‐CV), using the scikit‐learn library [48, 49, 50]. Through this process, we identified seven descriptors with the highest predictive value: *AATS6i* and *ATSC7m* (2D autocorrelation descriptors encoding the spatial distribution of atomic properties such as mass and polarizability), *VE1_DzZ* (a Barysz matrix descriptor capturing electronic and topological information), three Burden‐modified eigenvalues (*SpMax2_Bhm*, *SpMax1_Bhv*, and *SpMax2_Bhv*), and *CrippenLogP*, which estimates lipophilicity. Overall, the selected descriptors reflect molecular features essential for ligand–target interactions, such as shape complementarity, electrostatic properties, and hydrophobicity. The reduced descriptor set was finally used to train the final SVM model. Hyperparameters were optimized via grid search using scikit‐learn's GridSearchCV, resulting in the selection of *C* = 1.0 and *γ* = 1.0. The model demonstrated strong generalization performance, achieving 88% classification accuracy and 75% precision on an independent test set. In this context, *C* controls the trade‐off between maximizing the margin and minimizing classification errors, while *γ* defines the influence of individual data points on the decision boundary. Careful optimization of these parameters was essential to avoid overfitting given the limited dataset size, and to ensure robust generalization of the model.

### 4.2 MST Experiments

The binding of small molecules to the SARS‐CoV‐2 Mpro enzyme was assessed using the Monolith NT.115 instrument (NanoTemper Technologies GmbH, Munich, Germany). Recombinant His‐tagged SARS‐CoV‐2 Mpro (GeneTex, Irvine, CA, USA) was fluorescently labeled using the His‐Tag Labeling Kit RED‐tris‐NTA 2nd Generation (MO‐L018, NanoTemper Technologies GmbH, Munich, Germany) for 30 min at room temperature. A fixed concentration of red‐labeled SARS‐CoV‐2 Mpro (35 nM) was mixed with each of 180 small molecules at a fixed concentration (50 µM) to perform a binding check, using the “Expert Mode” of the MO. Control v1.6 software. The β‐amido boronic acid compound **3ea** was included as a positive control [35]. The binding was determined by monitoring compound concentration‐dependent changes in the normalized fluorescence (Fnorm), focusing on the 2.5‐second time point in all binding assays. A response amplitude (RA), defined as the Fnorm difference between the ligand‐bound and unbound states of SARS‐CoV‐2 Mpro, was considered indicative of binding when RA ≥ 1.5. The compounds that tested positive in the binding check assay underwent further assessment through the binding affinity experiment on the SARS‐CoV‐2 Mpro enzyme to generate a complete *K*_d_ curve. Initially, the protein was labeled using the same protocol, and then a fixed concentration of labeled SARS‐CoV‐2 Mpro (35 nM) was mixed with 16 1:1 serial dilutions of small molecules, ranging in concentration from 250 µM to 7.6 nM. Throughout both binding check and binding affinity MST experiments, the enzyme and ligands were incubated for 15–50 min at room temperature, employing a medium MST power (40%) and an excitation power of 80% at 25°C, and using standard capillaries (NanoTemper Technologies GmbH, München, Germany). Both interacting species were dissolved in PBS‐T buffer (phosphate‐buffered saline + 0.05% Tween 20) from NanoTemper Technologies (GmbH, München, Germany) and 2.5% dimethyl sulfoxide (DMSO) for molecular biology (Product No. D8418; Sigma‐Aldrich, Saint Louis, USA). The Monolith software MO. Affinity Analysis v2.3 was used to generate the full analysis report, while the figures were generated using GraphPad Prism software v8.0.2 (GraphPad, Boston, USA).

### 4.3 Protein Expression and Purification of SARS‐CoV‐2 Mpro

The protein was expressed and purified as described previously [51]. Briefly, the pE‐SUMO vector (Kindly provided by Prof. Dr. Harald Schwalbe, University of Frankfurt), encoding the SARS‐CoV‐2 Mpro protein with a hexahistidine (His_6_) tag attached to a small ubiquitin‐like modifier (SUMO) tag at the N‐terminus, was transformed into competent *Escherichia coli* (E. coli) BL21 Gold (DE3) cells (Agilent Technologies, Santa Clara, CA, USA). The cells were cultured at 37°C with shaking at 160 rpm in Luria Broth (LB) medium supplemented with 100 µg/mL ampicillin. Overexpression was induced by adding 0.2 mM isopropyl‐β‐d‐thiogalactopyranosid (IPTG), and the culture was incubated overnight at 18°C. The cells were then harvested by centrifugation at 4700 rpm for 1 h at 4°C. The resulting pellet was resuspended in 100 mL of lysis buffer (50 mM NaPi pH 7.5, 300 mM NaCl, 5 mM imidazole, 5% (v/v) glycerol, 10 mM β‐ME), which was supplemented with RNase, DNase, and lysozyme. The suspension was sonicated for 10 cycles of 30 s at 60% power on ice, with 10‐s pauses between each pulse. To remove insoluble material, the lysate was centrifuged at 20,000 rpm for 1 h at 4°C.

The protein purification was carried out using an ÄKTA start protein purification system (GE Healthcare, Chicago, IL, USA). The supernatant was loaded onto a HisTrap HP 5 mL column (Cytiva Europe GmbH, Freiburg im Breisgau, Germany), which was pre‐equilibrated with the lysis buffer. The column was washed with five column volumes (CV) of the same buffer. Elution was achieved by applying a linear gradient of elution buffer (50 mM NaPi (pH 7.5), 300 mM NaCl, 500 mM imidazole, 5% (v/v) glycerol, 10 mM β‐ME). To exchange the elution buffer, the solution was passed through an Amicon Ultra‐15 Centrifugal filter (Millipore, Billerica, MA, USA) with a 10 kDa cutoff, replacing it with Ulp‐1 cleavage buffer (50 mM NaPi pH 7.0, 300 mM NaCl, 10 mM β‐ME, 5% (v/v) glycerol). For tag removal, 250 units of His_6_‐tagged Ulp‐1 protease (Sigma‐Aldrich, St. Louis, MO, USA) were added, and the reaction proceeded overnight at 4°C. The mixture was loaded onto a HisTrap HP 5 mL column, collecting the flow‐through to remove the Ulp‐1 protease and the tags. Lastly, the protein underwent size exclusion chromatography (SEC) on a HiLoad 16/600 Superdex 75 pg column (Cytiva Europe GmbH, Freiburg im Breisgau, Germany), pre‐equilibrated with SEC buffer (25 mM NaPi pH 7.5, 150 mM NaCl, 2 mM DTT). Afterward, the protease was exchanged into 10% (v/v) glycerol, aliquoted, flash frozen in liquid nitrogen, and stored at –80°C until further use.

### 4.4 Enzymatic Assays

Inhibitory activities of the compounds against SARS‐CoV‐2 Mpro were determined as described previously by cleavage of a fluorescence resonance energy transfer (FRET) peptide substrate (Dabcyl‐KTSAVLQSGFRKME‐Edans), purchased from Genescript (New Jersey, USA) [52].

Fluorescence measurements were carried out in white flat‐bottom 96‐well plates (Greiner Bio‐One, Kremsmünster, Upper Austria) using a Tecan Spark 10M plate reader (Tecan Group Ltd., Männedorf, Switzerland). Each well contained a final volume of 200 µL, consisting of 180 µL assay buffer (20 mM TRIS‐HCl pH 7.5, 20 mM NaCl, 0.1 mM EDTA, 1 mM DTT), 5 µL enzyme solution (final concentration 75 nM), 10 µL inhibitor in DMSO (final concentration 20 µM) or pure DMSO as a negative control, and 5 µL substrate solution in DMSO (final concentration 25 µM). The reaction was monitored for 10 min at 25°C, with fluorescence readings taken at 30‐s intervals. The substrate was excited at 335 nm, and fluorescence emission was recorded at 493 nm. The remaining enzymatic activity was determined by comparing the substrate hydrolysis rate in reaction mixtures containing the inhibitors to that of the DMSO control. Only the first 5 min of the fluorescence curves (Supporting Information S1: Figure S3) were used for the calculation to obtain the linear part.

### 4.5 Computational Studies

The SARS‐CoV‐2 Mpro crystal structure used in this study was retrieved from the Protein Data Bank (PDB accession code: 6W63), in which the protein is bound to a potent broad‐spectrum non‐covalent inhibitor (namely, X77) [53]. Protein structure refinement was carried out using the “*Protein Preparation Wizard”* tool of Maestro software suite (Release 2025‐1, Schrödinger LLC, New York, NY, USA). The protocol included: (i) addition of hydrogen atoms and reconstruction of any missing side chains; (ii) optimization of hydrogen‐bonding networks; and (iii) restrained energy minimization of all atoms to a maximum RMSD value of 0.3 Å, applying the OPLS4 force field. Co‐crystallized ligands were removed before preparation. Binding site prediction was performed using the “*SiteMap*” tool of Maestro software suite (Release 2025‐1, Schrödinger LLC, New York, NY, USA), considering all protein atoms and employing default parameters. Compounds **7**, **8**, and **9** were manually designed and prepared using the “*LigPrep*” tool of Maestro software suite (Release 2025‐1, Schrödinger LLC, New York, NY, USA), applying the OPLS4 force field and Epik Classic for ionization state prediction at a pH of 7 ± 2. The docking grid was generated based on the residues defining the Site1 and Site2 pockets, namely Gln107, Pro108, Gln110, Asn151, Asp153, Tyr154, Val202, Asn203, Glu240, His246, Ile249, Thr292, Phe294, Asp295, and Arg298. The dimensions of the bounding box were set to encompass both binding sites. Docking calculations were performed using the “*Glide*” tool available in Maestro software suite (Release 2025‐1, Schrödinger LLC, New York, NY, USA) with the XP mode enabled. The number of poses to generate per ligand was set to 500, and the energy threshold for rejecting minimized poses was set to 0.5 kcal/mol. The reliability of the docking calculations was assessed by performing a self‐docking calculation of AT7519 compound, in which the top‐ranked binding pose was compared with its crystallographic conformation (PDB ID: 7AGA [37]), yielding an excellent agreement with an RMSD of 2.16 Å (Supporting Information S1: Figure S11). The three best docking poses of each compound were carefully examined to assess potential differences in binding modes, and the RMSD of the ligands were calculated after aligning the protein backbone atoms (Supporting Information S1: Figure S12). Since each compound essentially exhibited a single predominant binding pose, only the top‐ranked pose of each compound/Mpro complex was immersed in an orthorhombic box of TIP3P water molecules using the “*System Builder*” tool in Maestro software suite (Release 2025‐1, Schrödinger LLC, New York, NY, USA), with box boundaries set to 10 Å from the protein surface. System neutrality was achieved by adding the appropriate number of counterions. Following solvation and energy minimization, each system underwent to three independent 500 ns MD simulations using the “*Desmond*” algorithm implemented in the Maestro software suite (Release 2025‐1, Schrödinger LLC, New York, NY, USA) under constant conditions of 300 K and 1 atm. The protein/ligand stability (Cα RMSD plots) throughout the simulation was evaluated using the “*Simulation Interactions Diagram*” tool available in Maestro software suite (Release 2025‐1, Schrödinger LLC, New York, NY, USA). Conformational clustering of ligand structures obtained from MD simulations was carried out using the GROMOS algorithm by Daura et al. [54], as implemented in the GROMACS software package (version 2024.3) [55]. Several clustering trials were conducted to determine an optimal RMSD cutoff, enabling clear discrimination among ligand conformations while reducing the prevalence of isolated (singleton) clusters (Supporting Information S1: Table S3).

## Funding

The authors received no specific funding for this work.

## Conflicts of Interest

The authors declare no conflicts of interest.

## Supporting information

SuppInfo_final.

ArchPharm_SupplMat_InChI.

## Data Availability

Data will be made available on request.
