# Supplementary material for: Support Vector Machine Identification of Small Molecule Binders to an Understudied Allosteric Site of SARS‐CoV‐2 Mpro for Next‐Generation PROTAC‐Based Therapeutics
Source: Arch Pharm (Weinheim). 2025 Dec 13;358(12):e70169. doi: 10.1002/ardp.70169 (PMC12701685; doi:10.1002/ardp.70169)
Supplement: Supplementary file 1 — SuppInfo_final. [file ARDP-358-e70169-s002.pdf]

Supporting Information

Support Vector Machine Identification of Small Molecule Binders to an Understudied Allosteric Site of SARS-Cov-2 Mpro for Next-Generation PROTAC-Based Therapeutics

Enrico Mario Alessandro Fassi[1], Nedra Mekni[2,*], Marco Albani[1], Sabine Maehrlein[3], Annabelle Carolin Weldert[3], Tanja Schirmeister[3], Thierry Langer[2] and Giovanni Grazioso[1]

[1] Department of Pharmaceutical Sciences, Università degli Studi di Milano, Via L. Mangiagalli 25, 20133 Milano, Italy.

[2] Department of Pharmaceutical Sciences, Division of Pharmaceutical Chemistry, University of Vienna, Josef-Holaubek-Platz 2, 1090.

[3] Department of Medicinal Chemistry, Institute of Pharmaceutical and Biomedical Sciences, Johannes Gutenberg-University, 55128, Mainz, Germany.

* Corresponding author: Nedra Mekni (meknene@gmail.com)

Table S1. Chemical structures of the 17 compounds successfully tested positive for the binding check MST experiments and selected for further assessment in the binding affinity experiments.

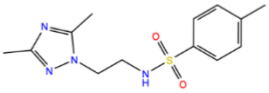 Compound 1	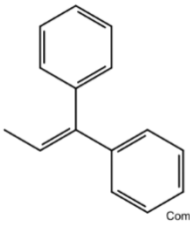 Compound 2	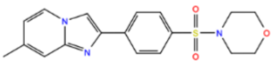 Compound 3
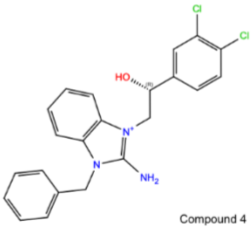 Compound 4	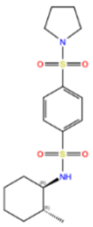 Compound 5	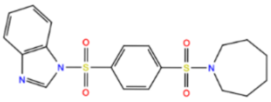 Compound 6
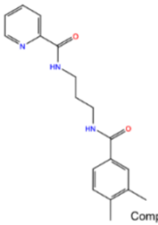 Compound 7	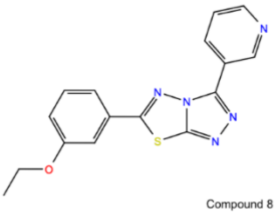 Compound 8	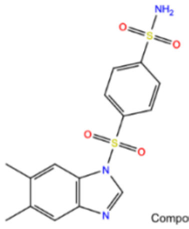 Compound 9
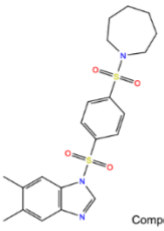 Compound 10	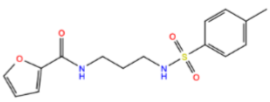 Compound 11	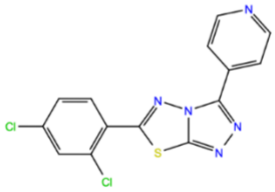 Compound 12

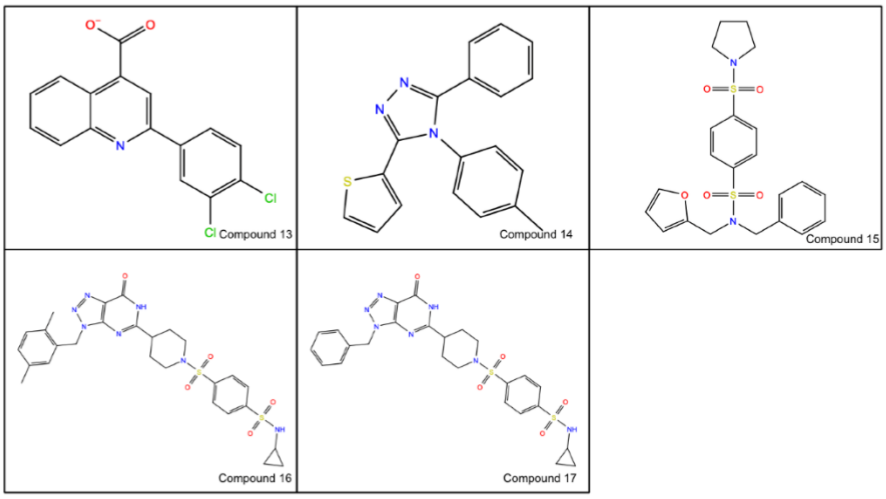

Table S2. Summary table of the MST experiments performed using a fixed concentration of SARS-CoV-2 Mpro (35 nM) and scaling concentration of the compounds **7**, **8** and **9**. Two independent experiments were performed to compute the K_d value.

Compound	MST Power	Exc. Power	Temp.	[Ligand] Range	Time	RA	SNR	K_d (µM)
**7**	40%	80%	25 °C	31.3 µM - 7.6 nM	5 s	3.4	13.6	2.8 ± 0.9
**8**	40%	80%	25 °C	250 µM - 7.6 nM	5 s	4.7	13.1	23.9 ± 7.4
**9**	40%	80%	25 °C	250 µM - 7.6 nM	2.5 s	2.7	8.8	39.0 ± 23.4

RA = Response Amplitude, SNR = Signal-to-Noise Ratio.

Figure S3. Results of the cluster analysis applying different cut-off levels. Values of the three main clusters are expressed as percentages (%). In bold is highlighted the chosen cut-off, with the corresponding color used in the article for the compounds.

Compound 7					Compound 8					Compound 9				
cut-off	N°	1°	2°	3°	cut-off	N°	1°	2°	3°	cut-off	N°	1°	2°	3°
1.4 Å	33	25.9	9.8	9.4	0.4 Å	121	9.9	8.9	8.2	0.4 Å	47	70.9	4.9	3.3
1.6 Å	19	33.7	16.0	13.3	0.6 Å	30	30.2	24.1	12.3	0.6 Å	17	80.9	4.8	4.2
1.8 Å	10	41.9	26.4	14.1	0.8 Å	12	48.5	35.4	7.3	0.8 Å	13	81.6	6.0	4.2
**2.0 Å**	**6**	**48.7**	**38.5**	**8.2**	**1.0 Å**	**8**	**64.8**	**21.6**	**8.6**	**1.0 Å**	**12**	**85.5**	**6.8**	**3.6**
2.2 Å	4	67.0	17.2	15.0	1.2 Å	4	86.5	10.5	2.5	1.2 Å	4	95.4	3.2	1.2
2.4 Å	4	88.3	9.4	1.2	1.4 Å	2	89.0	11.0	/	1.4 Å	3	99.3	0.4	0.3

N° = Number of total clusters.

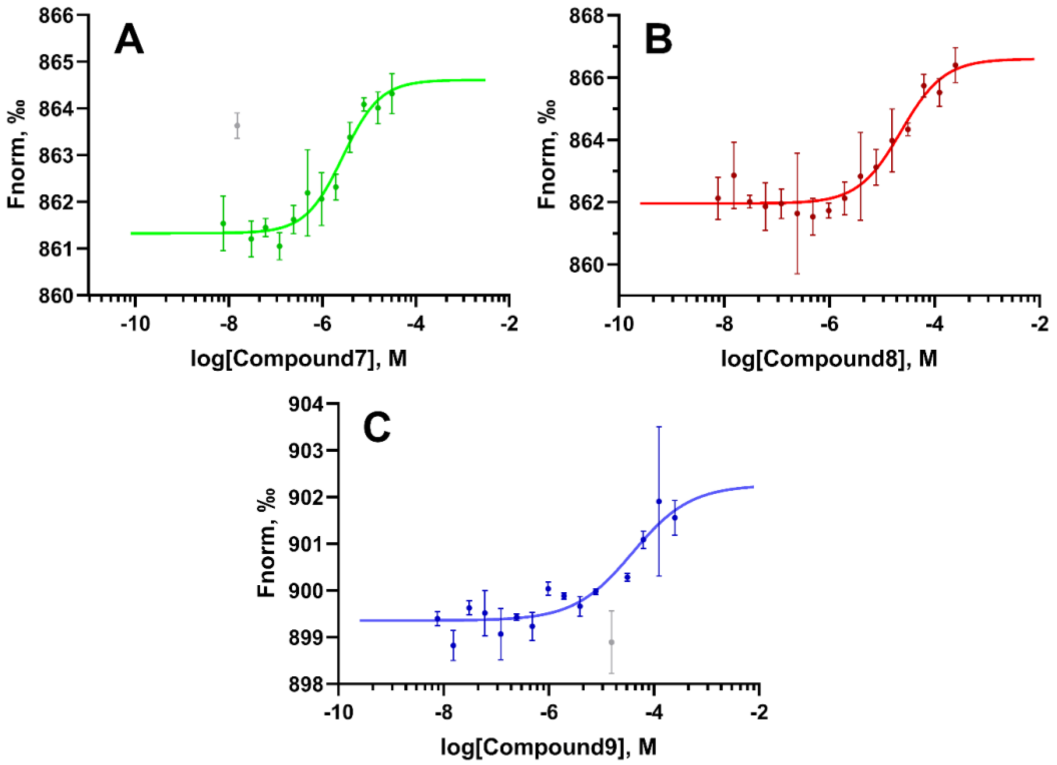

Figure S1. MST curves acquired by SARS-CoV-2 Mpro incubated with different concentrations of compounds **7** (A), **8** (B) and **9** (C) using the Monolith NT.115 instrument. In the case of **7** and **9**, the two points evidenced in grey (corresponding to the concentration of 15.2 nM and 15.6 µM, respectively) were excluded by fitting, since they are clear outliers.

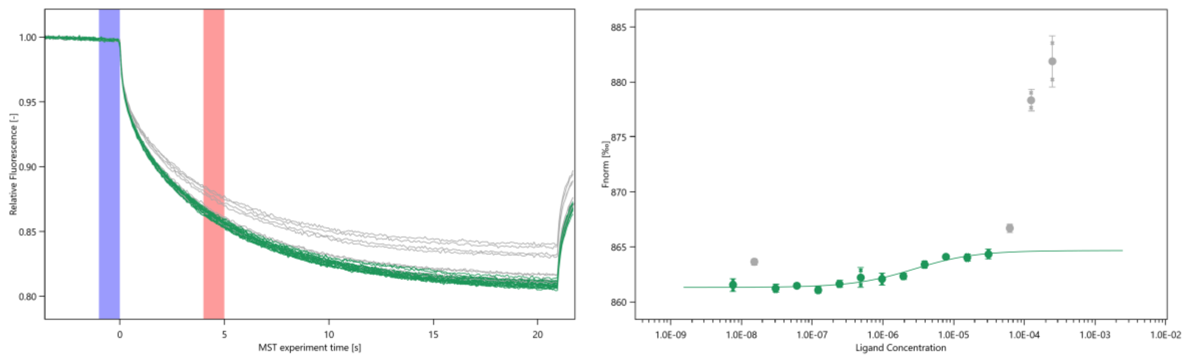

Figure S2. Full MST trace (left) and MST binding curve (right) of compound **7** including the first three concentration points (250, 125, and 62.5 µM; highlighted in grey) that were omitted from fitting, as they likely reflect a distinct, low-affinity interaction evident in the high-micromolar range.

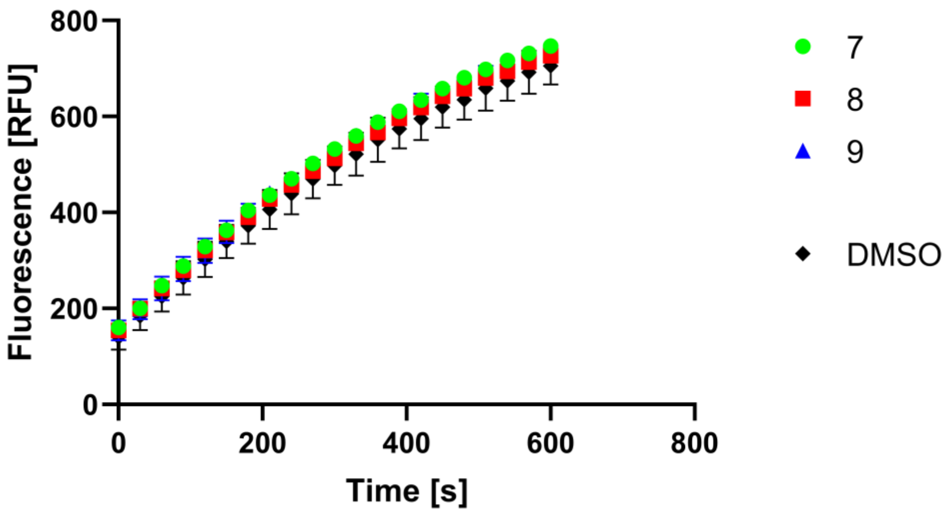

Figure S3. Fluorescence curves used to determine inhibitory activity. The negative control (DMSO) is shown in black, compound **7** in green, compound **8** in red, and compound **9** in blue.

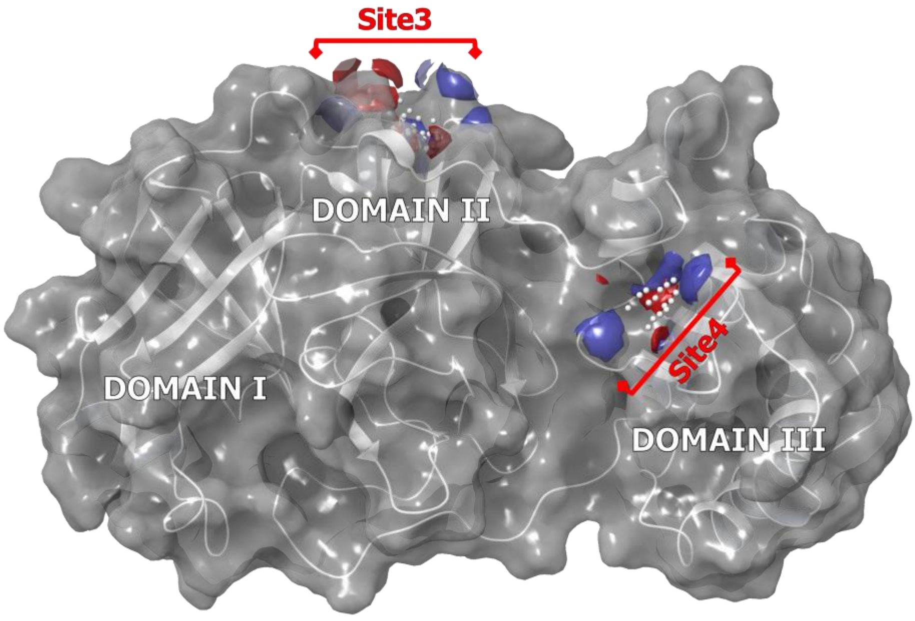

Figure S4. Representation of the other two less promising allosteric binding sites (Site3 and Site4) identified with SiteMap located within Domain II and Domain III of SARS-CoV-2 Mpro, respectively.

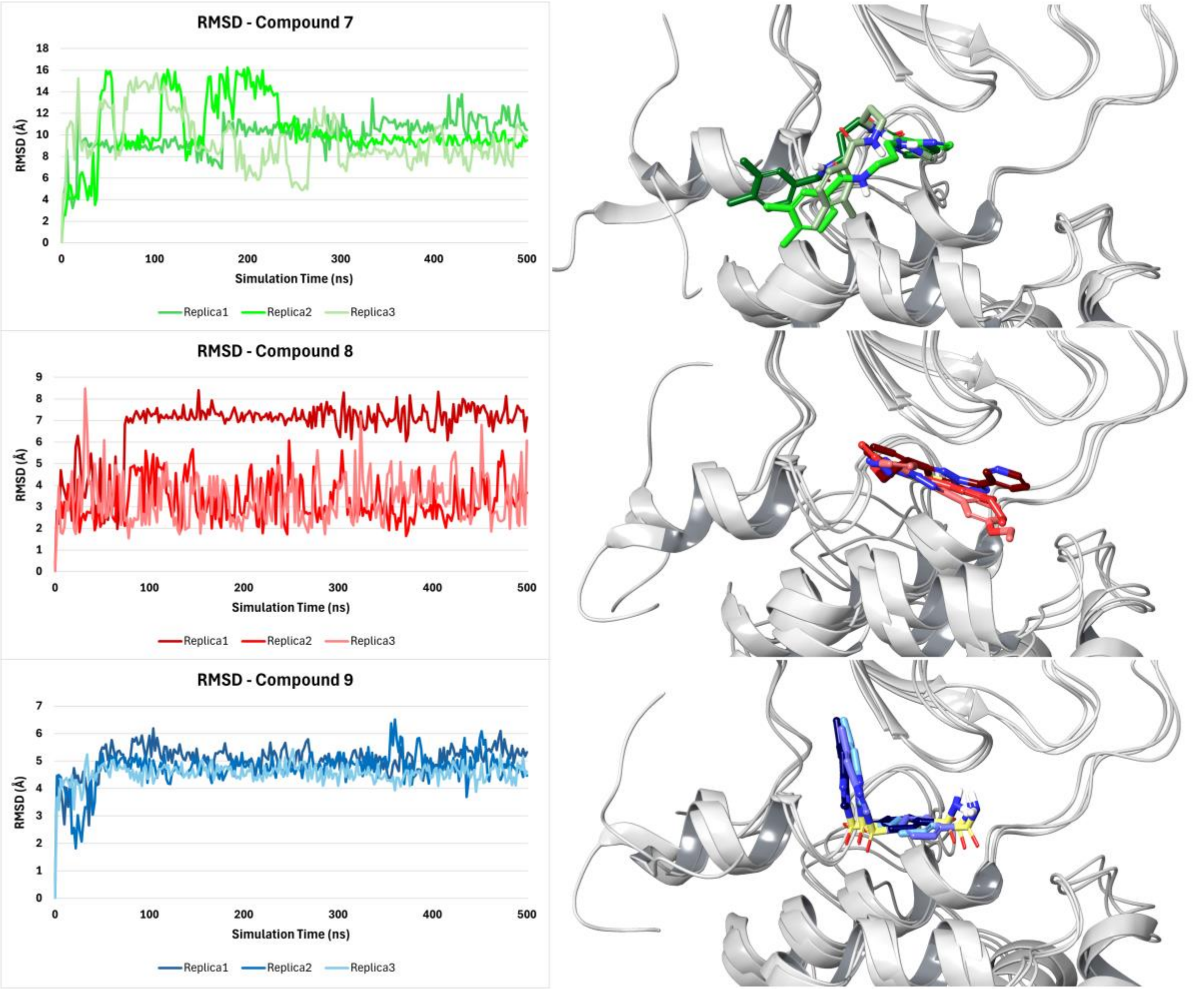

Figure S5. (Left) Ligand heavy atoms RMSD after aligning each complex to the protein backbone, calculated over three independent MD replicas. (Right) Representative ligand conformations after equilibration during the MD simulations; the color of each structure corresponds to its respective RMSD trace.

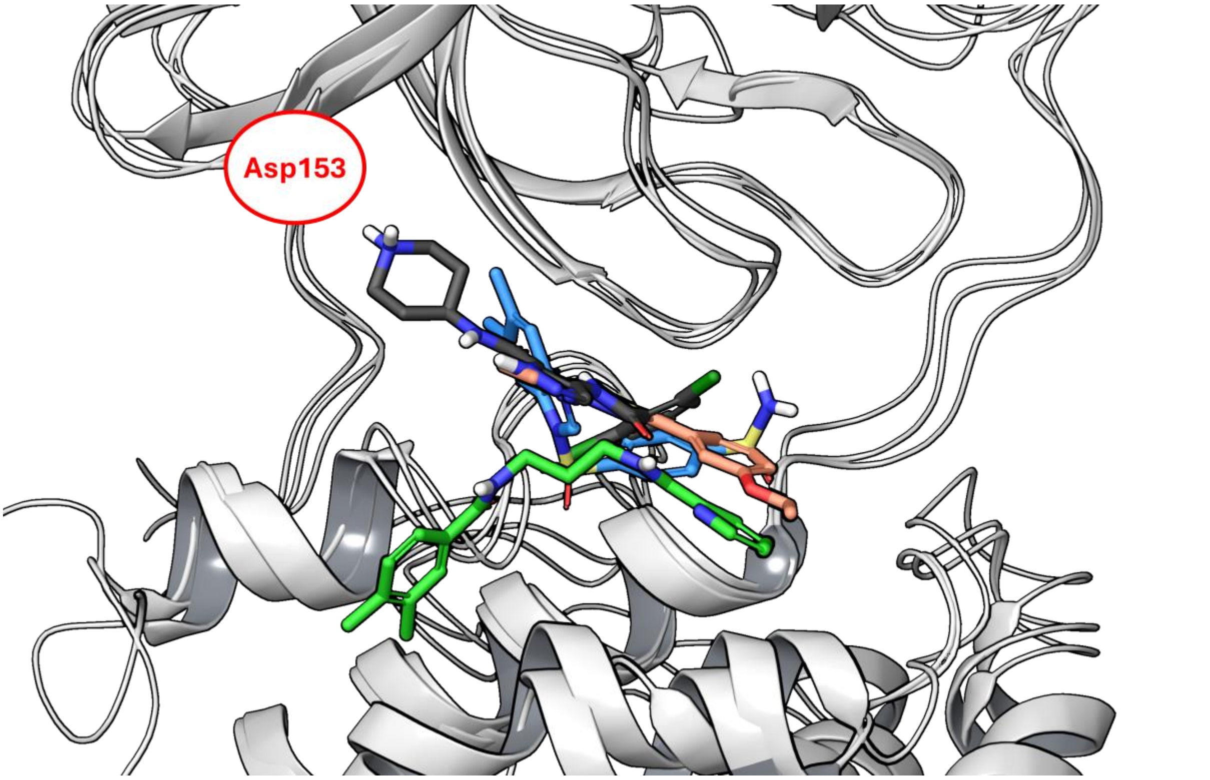

Figure S6. Superposition of the crystal structure of AT7519 (dark grey sticks, PDB code 7AGA) with the representative structures from the most populated cluster of compounds **7**, **8** and **9**. The position of the key residue Asp153, critical for catalytic inhibition of SARS-CoV-2 Mpro by AT7519, is highlighted.

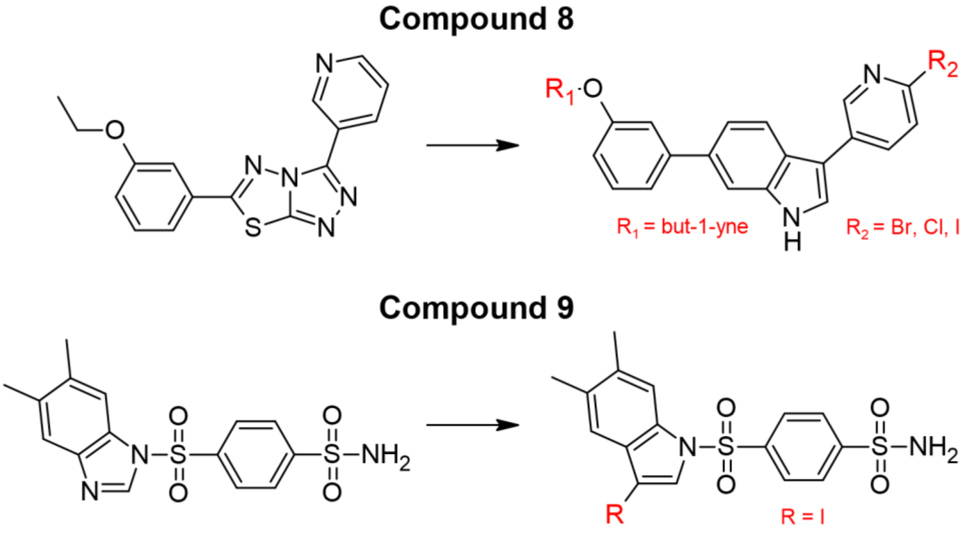

Figure S7. (Left) Original structures of compounds **8** and **9**. (Right) Proposed optimal linker positions for rational design of potential PROTACs targeting SARS-CoV-2 Mpro, indicated by red "R" labels.

2.7 Rational design of PROTACs (continued).

Compound **8** represents a peculiar case, as it possesses two potential solvent-exposed positions where the addition of a linker for PROTAC formation could be envisioned (R_1 and R_2, **Figure**

S7). To improve the synthetic feasibility of the compound we suggest synthesizing the indole analogue (**Figure S7**), which retain the spatial orientation of functional groups responsible for the interaction with the enzyme. Here, for the synthesis of PROTACs in which the degrader moiety corresponds to the substituent R_1 (**Figure S7**), an alkyne could replace the ethyl substituent attached to the benzene ring, enabling a subsequent Huisgen 1,3-dipolar cycloaddition between an azide and an alkyne to form the triazole linkage (Click chemistry). According to the CAS SciFinder® retrosynthetic analysis, the preparation of the compound ready for linker installation in this configuration is predicted to give a 24% yield with an estimated cost of $231 per 100 grams. Starting from commercially available 6-chloroindole, the compound 3,6-dichloro-1H-indole was synthesized via a halogenation reaction. The latter was subsequently converted to (6-chloro-1H-indol-3-yl)boronic acid and subjected to Suzuki-Miyaura cross-coupling with commercially available 3-chloro-pyridine to yield 6-chloro-3-(pyridin-3-yl)-1H-indole **(a)**. In parallel, the commercially available compounds 3-chlorophenol and propargyl bromide react to afford 1-chloro-3-(2-propyn-1-yloxy)benzene (also commercially available). The latter is then converted into the corresponding boronic acid, B-[3-(2-propyn-1-yloxy)phenyl]boronic acid **(b)**. Finally, compound **(a)** and compound **(b)** are coupled to yield the final derivative, suitably functionalized for the ensuing Huisgen cycloaddition to attach the linker. The retrosynthesis scheme is reported in **Figure S8**.

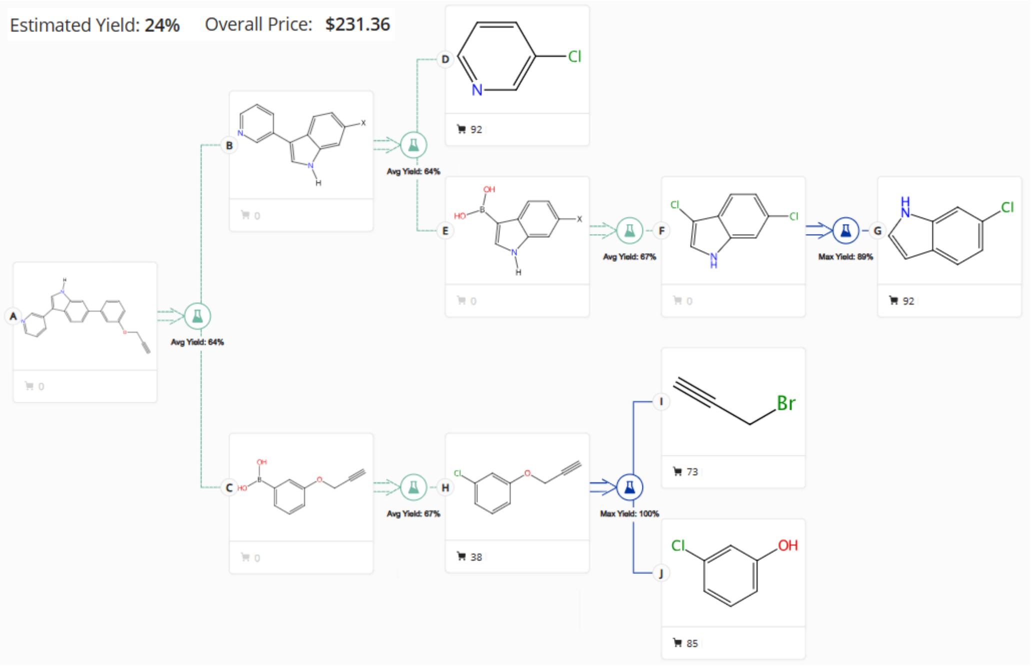

Figure S8. Retrosynthetic analysis of compound **8** intermediate for PROTACs featuring the R1-derived degrader moiety. The black cart indicates that the product is commercially available and the number of vendors. The blue reaction symbol indicates that the reaction has been already published in literature, while the green one that the reaction is possible, although do not already accomplish using the specific reagents. The scheme was generated through the “retrosynthesis tool” available on CAS-SciFinder web platform.

On the other hand, for the synthesis of PROTACs in which the degrader moiety corresponds to the substituent R_2 (**Figure S7**), halogenation of the pyridine moiety with iodine, bromine, or chlorine was considered to enable subsequent linker installation via Sonogashira cross-coupling. The estimated yields for all three halides were comparable, with iodine providing an estimated yield of 27%, and both bromine and chlorine around 24%. However, bromine is more promising in terms of cost, with an estimated price of $298 per 100 g, compared to $308 for chlorine and $443 for iodine per 100 g. This cost-efficiency, combined with competitive yields, supports bromine as a favorable choice for further optimization in PROTAC linker installation via Sonogashira cross-coupling. Starting from commercially available 6-chloroindole, the reaction was performed as previously described for R_1, except that it was reacted with 2-bromo-5-chloropyridine to afford 3-(6-bromopyridin-3-yl)-6-chloro-1H-indole. This intermediate underwent a second Suzuki-Miyaura coupling with 3-ethoxyphenylboronic acid to afford the target compound, 3-(6-bromopyridin-3-yl)-6-(3-ethoxyphenyl)-1H-indole, which is now poised for Sonogashira cross-coupling reaction. The retrosynthesis scheme is reported in **Figure S9**.

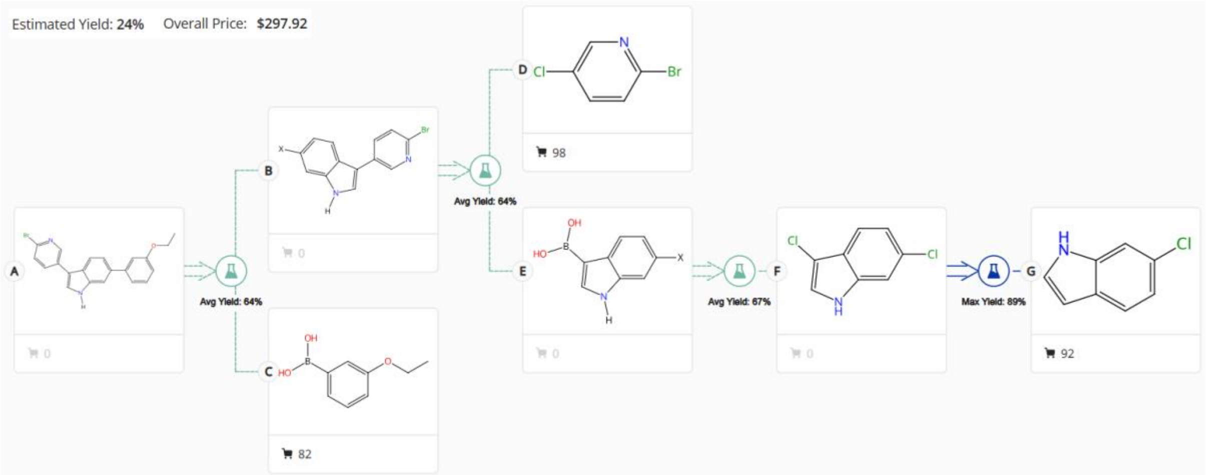

Figure S9. Retrosynthetic analysis of compound **8** intermediate for PROTACs featuring the R_2-derived degrader moiety. The black cart indicates that the product is commercially available and the number of vendors. The blue reaction symbol indicates that the reaction has been already published in literature, while the green one that the reaction is possible, although do not already accomplish using the specific reagents. The scheme was generated through the "retrosynthesis tool" available on CAS-SciFinder web platform.

Regarding compound **9**, the most suitable site to attach the PROTAC moiety is the nitrogen atom at position 3 of the benzimidazole ring. We therefore propose substituting this nitrogen atom with a carbon atom to generate an indole scaffold with an aryl halide (iodo-, bromo-, or chloro-substituted derivatives), thereby facilitating linker installation via Sonogashira cross-coupling reaction. Ultimately, only iodine showed acceptable estimated yield and cost values (23% and $364 per 100 grams, respectively) whereas the other two halogens exhibited yields below 20% and costs exceeding

$500 per 100 grams. Starting from commercially available 5,6-dichloroindole, iodination affords 5,6-dichloro-3-iodo-1H-indole, which is also commercially available. The latter reacts with commercially available methylmagnesium chloride (MeMgCl) to yield 3-iodo-5,6-dimethyl-1H-indole **(c)**. In parallel, commercially available 4-aminobenzenesulfonamide undergoes sulfonylation to form 4-(aminosulfonyl)benzenesulfonyl chloride **(d)**. Coupling of compounds **(c)** + **(d)** permit to obtain the target compound 4-((3-iodo-5,6-dimethyl-1H-indol-1-yl)sulfonyl)benzenesulfonamide through a nucleophilic substitution reaction. The synthesis scheme is reported in **Figure S10**.

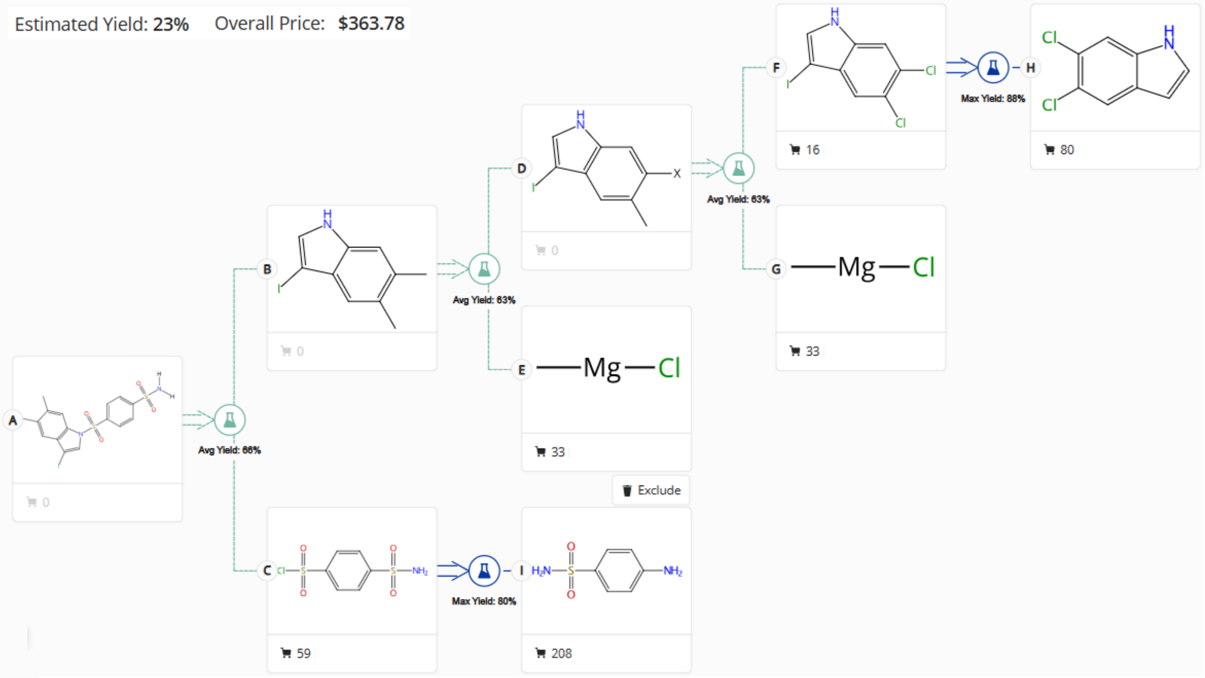

Figure S10. Retrosynthetic analysis of compound **9** intermediate relevant to PROTAC synthesis. The black cart indicates that the product is commercially available and the number of vendors. The blue reaction symbol indicates that the reaction has been already published in literature, while the green one that the reaction is possible, although do not already accomplish using the specific reagents. The scheme was generated through the “retrosynthesis tool” available on CAS-SciFinder web platform.

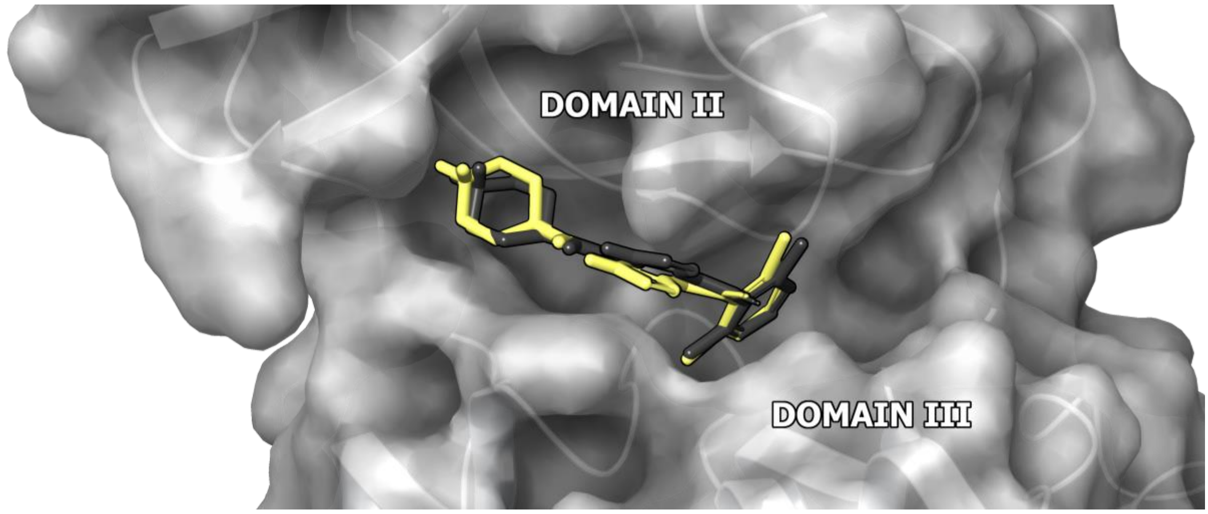

Figure S11. Comparison between the self-docking pose of AT7519 (yellow sticks) and its crystallographic pose (black sticks, PDB ID: 7AGA) at the interface between Domain II and Domain III of SARS-CoV-2 Mpro (represented as a light grey surface and cartoon representation).

Compound 7	Docking Score (kcal/mol)	RMSD (Å)
Pose #1	−3.440	reference
Pose #2	−3.115	1.02
Pose #3	−2.945	3.42

Compound 8	Docking Score (kcal/mol)	RMSD (Å)
Pose #1	−3.279	reference
Pose #2	−3.127	0.45
Pose #3	−3.093	1.03

Compound 9	Docking Score (kcal/mol)	RMSD (Å)
Pose #1	−2.026	reference
Pose #2	−1.703	0.25
Pose #3	−1.611	0.92

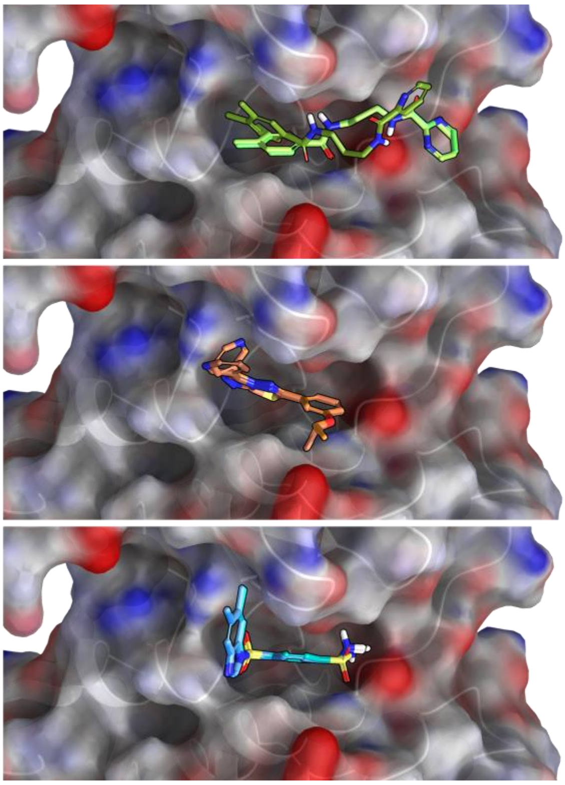

Figure S12. (Left) Docking scores for the three top-ranked poses of each compound, along with the RMSD of the compounds relative to their best-scoring pose, calculated after aligning the protein backbone atoms. (Right) Structural representation of the top three docking poses for each compound (sticks) bound to the SARS-CoV-2 Mpro (light grey surface and ribbons). The solvent-accessible surface of the protein is color-coded according to atomic partial charges, with blue indicating positively charged regions and red indicating negatively charged residues.
