# Supplementary material for: Support Vector Machine Identification of Small Molecule Binders to an Understudied Allosteric Site of SARS‐CoV‐2 Mpro for Next‐Generation PROTAC‐Based Therapeutics
Source: Arch Pharm (Weinheim). 2025 Dec 13;358(12):e70169. doi: 10.1002/ardp.70169 (PMC12701685; doi:10.1002/ardp.70169)
Supplement: Supplementary file 2 — ArchPharm_SupplMat_InChI. [file ARDP-358-e70169-s001.doc]

**Supplemental Material: Novel Compounds and Biological Screening Results**

**Support Vector Machine Identification of Small Molecule Binders to an Understudied Allosteric Site of SARS-Cov-2 Mpro for Next-Generation PROTAC-Based Therapeutics**

Enrico Mario Alessandro Fassi^1^, Nedra Mekni^2^,*, Marco Albani^1^, Sabine Maehrlein^3^, Annabelle Carolin Weldert^3^, Tanja Schirmeister^3^, Thierry Langer^2^, and Giovanni Grazioso^1^

^1^ Department of Pharmaceutical Sciences, Università degli Studi di Milano, Via L. Mangiagalli 25, 20133 Milano, Italy.

^2^ Department of Pharmaceutical Sciences, Division of Pharmaceutical Chemistry, University of Vienna, Josef-Holaubek-Platz 2, 1090.

^3^ Department of Medicinal Chemistry, Institute of Pharmaceutical and Biomedical Sciences, Johannes Gutenberg-University, 55128, Mainz, Germany.

* Corresponding author: Nedra Mekni ([nedram19@univie.ac.at](mailto:nedram19@univie.ac.at))

| **Compound No.** | **InChI** | **Biological Activity (*K*_D_)^a^** |
| --- | --- | --- |
| **7** | InChI=1S/C18H21N3O2/c1-13-7-8-15(12-14(13)2)17(22)20-10-5-11-21-18(23)16-6-3-4-9-19-16/h3-4,6-9,12H,5,10-11H2,1-2H3,(H,20,22)(H,21,23) | 2.8 ± 0.9 µM |
| **8** | InChI=1S/C16H13N5OS/c1-2-22-13-7-3-5-11(9-13)15-20-21-14(18-19-16(21)23-15)12-6-4-8-17-10-12/h3-10H,2H2,1H3 | 23.9 ± 7.4 µM |
| **9** | InChI=1S/C15H15N3O4S2/c1-10-7-14-15(8-11(10)2)18(9-17-14)24(21,22)13-5-3-12(4-6-13)23(16,19)20/h3-9H,1-2H3,(H2,16,19,20) | 39.0 ± 23.4 µM |

^a^ K_d_ values of compounds who displayed a clear K_d_ curve in MST.
